# Demonstration of a novel Xp22.2 microdeletion as the cause of familial extreme skewing of X‐inactivation utilizing case‐parent trio SNP microarray analysis

**DOI:** 10.1002/mgg3.378

**Published:** 2018-02-28

**Authors:** Jane A. Mason, Hnin T. Aung, Adayapalam Nandini, Rickie G. Woods, David J. Fairbairn, John A. Rowell, David Young, Rachel D. Susman, Simon A. Brown, Valentine J. Hyland, Jeremy D. Robertson

**Affiliations:** ^1^ Queensland Haemophilia Centre Department of Haematology and Cancer Care Royal Brisbane and Women's Hospital Brisbane QLD Australia; ^2^ Department of Cytogenetics Pathology Queensland Royal Brisbane and Women's Hospital Brisbane QLD Australia; ^3^ Department of Molecular Genetics Pathology Queensland Royal Brisbane and Women's Hospital Brisbane QLD Australia; ^4^ Genetic Health Queensland Royal Brisbane and Women's Hospital Brisbane QLD Australia; ^5^ Department of Haemophilia and Haematology Lady Cilento Children's Hospital Brisbane QLD Australia

**Keywords:** female, genetics, hemophilia, pediatric, X inactivation

## Abstract

**Background:**

We report a kindred referred for molecular investigation of severe hemophilia A in a young female in which extremely skewed X‐inactivation was observed in both the proband and her clinically normal mother.

**Methods:**

Bidirectional Sanger sequencing of all *F8* gene coding regions and exon/intron boundaries was undertaken. Methylation‐sensitive restriction enzymes were utilized to investigate skewed X‐inactivation using both a classical human androgen receptor (HUMARA) assay, and a novel method targeting differential methylation patterns in multiple informative X‐chromosome SNPs. Illumina Whole‐Genome Infinium microarray analysis was performed in the case‐parent trio (proband and both parents), and the proband's maternal grandmother.

**Results:**

The proband was a cytogenetically normal female with severe hemophilia A resulting from a heterozygous *F8* pathogenic variant inherited from her similarly affected father. No *F8* mutation was identified in the proband's mother, however, both the proband and her mother both demonstrated completely skewed X‐chromosome inactivation (100%) in association with a previously unreported 2.3 Mb deletion at Xp22.2. At least three disease‐associated genes (*FANCB*,*AP1S2,* and *PIGA*) were contained within the deleted region.

**Conclusions:**

We hypothesize that true “extreme” skewing of X‐inactivation (≥95%) is a rare occurrence, but when defined correctly there is a high probability of finding an X‐chromosome disease‐causing variant or larger deletion resulting in X‐inactivation through a survival disadvantage or cell lethal mechanism. We postulate that the 2.3 Mb Xp22.2 deletion identified in our kindred arose de novo in the proband's mother (on the grandfather's homolog), and produced extreme skewing of X‐inactivation via a “cell lethal” mechanism. We introduce a novel multitarget approach for X‐inactivation analysis using multiple informative differentially methylated SNPs, as an alternative to the classical single locus (HUMARA) method. We propose that for females with unexplained severe phenotypic expression of an X‐linked recessive disorder trio‐SNP microarray should be undertaken in combination with X‐inactivation analysis.

## 1 INTRODUCTION

It is well‐recognized that females who are obligate carriers of an X‐linked recessive disorder may rarely manifest clinical features consistent with a severe (“complete”) phenotype of the condition. This occurs through one of several known mechanisms, including overt cytogenetic abnormalities affecting the X‐chromosome (e.g. partial or complete monosomy X, Turner syndrome), compound heterozygosity or homozygosity for the disease‐causing variant, and endocrine disorders affecting the external genitalia (i.e. genetic males who are phenotypically female) (Puck & Willard, 1998). Extreme skewing of X‐inactivation is also an oft‐cited mechanism for the occurrence of a severe X‐linked recessive disorder in a cytogenetically normal female, although the underlying genetic basis for such skewing is rarely determined. Normally during early female embryogenesis, in order to achieve dosage compensation, one X‐chromosome in each cell is randomly and permanently inactivated in a process known as lyonization (Lyon, 1962). Typically in a female carrier of an X‐linked recessive disorder approximately equal portions of normal and mutant X‐chromosomes are inactivated, however, as the process is random, the X‐inactivation ratios observed in a population are distributed around a mean value (Belmont, 1996). Although in theory extreme skewing of X‐inactivation could occur through chance alone, the possibility that this phenomenon could be a familial trait inherited in a Mendelian fashion appears underrecognized when investigating females manifesting X‐linked disorders.

Linkage analysis has previously been undertaken to investigate large kindreds in which familial extreme skewing of X‐inactivation was discovered following the appearance of a female with a severe X‐linked recessive disorder (Pegoraro et al., 1997) Whilst such research provides highly valuable insight regarding the basis of familial skewing, it is clearly not feasible to undertake linkage analysis in a routine clinical setting, nor is this approach useful in the investigation of smaller kindreds. In most such kindreds reported in the literature the genetic basis for familial skewed X‐inactivation has not been established (Cau et al., 2006; Naumova et al., 1996; Renault et al., 2007). Discovery of the *XIST* gene in 1992 as the primary regulator of X‐chromosome inactivation represented a major step forward in the field (Belmont, 1996), however, pathogenic variants in *XIST* appear to be an uncommon cause of familial skewing.

Hemophilia A (MIM 306700) is a bleeding disorder caused by pathogenic variants in the *F8* gene (Xq28) resulting in reduced coagulation factor (F)VIII levels, and a bleeding phenotype that is inversely proportionate to residual FVIII activity. Severe hemophilia A occurs when the FVIII level is <0.01 IU/ml, and is inherited in a classical X‐linked recessive pattern (Oldenburg, Pezeshkpoor, & Pavlova, 2014). It has a number of features that make it an excellent prototype condition for the study of X‐inactivation. Firstly, the protein product of the *F8* gene is directly measurable in plasma, and can be precisely quantitated in routine coagulation laboratories. Secondly, with the advent of modern haemostatic replacement therapy (recombinant FVIII products), affected males typically survive to reproductive age and have normal fertility (Franchini & Mannucci, 2013). Thirdly, plasma concentration of FVIII is a direct reflection of liver (sinusoidal) synthesis therefore providing an accessible endoderm‐derived surrogate for assessment of skewed X‐inactivation in carrier females (Tuddenham, 2014).

## 2 MATERIALS AND METHODS

### 2.1 Ethical compliance

Ethical approval was obtained from the Royal Brisbane and Women's Hospital Human Research Ethics Committee (HREC/17/QRBW/332).

### 2.2 Study participants

**Figure 1:**
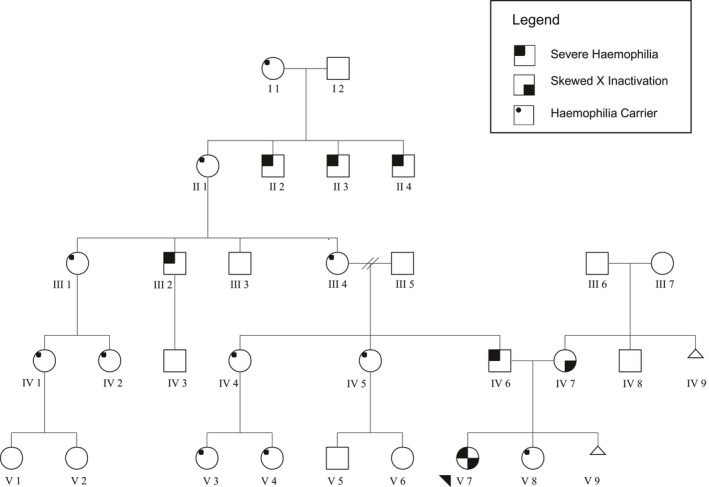
Severe hemophilia A and extremely skewed X‐inactivation kindred

The proband (Figure 1, V‐7), her father (Figure 1, IV‐6) and great uncle (Figure 1, III‐2) were known patients of our Haemophilia Centre. Informed consent was obtained from the adult family members who were formally enrolled in this study (Figure 1, IV‐7, IV‐6, III‐7).

### 2.3 Hemostasis investigations

Plasma FVIII levels were performed using a single stage APTT‐based system (ACL TOP^®^ Analyser, DG‐FVIII deficient plasma, Diagnostic Grifols, Passeig Fluvial, Spain). Von Willebrand Factor Antigen (VWF:Ag) and Ristocetin Cofactor (VWF:RCo) assays were performed using latex particle immune‐turbidometric assays (ACL TOP Analyser®, HemosIL reagents®, Instrumentation Lab, Bedford MA, USA). VWF:CB was performed using a commercial ELISA. Von Willebrand Factor Activity (VWF:Ac) was performed using a latex particle immune‐turbidometric assay (ACL TOP Analyser®, HemosIL reagent™, Instrumentation Lab, Bedford MA, USA).

### 2.4 DNA extraction

Patient DNA was extracted from whole blood using the Qiagen Symphony DSP DNA Midi kit on the QIASymphony SP extraction platform (Qiagen, Hilden, Germany). The quality and quantity of purified DNA was determined spectrophotometrically using the Nanodrop 8000 (Thermo Fisher Scientific, Waltham MA, USA).

### 2.5 Karyotyping

Specimens were collected in lithium heparin tubes and cultured for 48 hours in commercially available culture media (RPMI) at 37 degrees. Genial Genetics CellSprint automated suspension metaphase harvester was used to harvest cultured peripheral blood lymphocytes as per manufacturer's instructions. Fifteen G‐banded metaphases were then analysed per specimen for karyotyping.

### 2.6 Gene studies

The *F8* (Xq28) pathogenic variant spectrum in hemophilia A is diverse and includes single nucleotide variants (SNVs), short insertions and deletions (indels) and structural variants (SVs). The *F8* gene was screened for SNVs and indels by bidirectional Sanger sequencing of all *F8* gene coding regions and exon/intron boundaries. The intron 22 and intron 1 inversion SVs of *F8* cause about 45% and 3% of severe hemophilia A respectively (Oldenburg et al., 2014). Subcycling PCR was employed to detect and distinguish between the normal intron 22 (int22h1) and distal or proximal inversions (Kilian, Pospisil, & Hanrahan, 2006). PCR methodology was also used to detect and distinguish between the normal intron 1 (int1h‐1) and the extragenic homologue (int1h‐2) inverted DNA (Bagnall, Waseem, Green, & Giannelli, 2002). Multiplex Ligation Probe Amplification (MLPA) with probe mix P178 (MRC‐Holland, Amsterdam, Netherlands) was performed to screen for large deletions and duplication SVs in the *F8* gene.

Sequencing of the *XIST* (Xq13.2) gene was performed using Next Generation Sequencing (NGS) and included all coding and splicing regions and the upstream promotor region −1 to −33. (Fulgent Diagnostics, CA, USA).

### 2.7 X‐inactivation studies: classical (HUMARA) method

Methylation‐sensitive restriction enzymes *(HhaI* and *HpaII)* were used to investigate skewed X‐inactivation. PCR analysis was employed to amplify the highly polymorphic CAG trinucleotide repeat tract in exon 1 of the *AR* gene (Xq12) with and without prior methylation‐sensitive restriction enzyme digestion consistent with the method reported by Allen, Zoghbi, Moseley, Rosenblatt, and Belmont (1992). Digestion of unmethylated sites prevents PCR amplification and consequently differentiates a methylated allele from an unmethylated one. The relative proportion of X‐chromosome inactivation was then determined by calculating the ratios of the area under the curve of the two repeat sizes that correspond to the two AR alleles in females. We classified extremely skewed X‐chromosome inactivation as a ratio of greater than 95:5.

### 2.8 Single nucleotide polymorphism (SNP) microarrays

Illumina Whole‐Genome Infinium (CytoSNP 850K Array v1.1) microarray analysis was performed in the case‐parent trio (proband and both parents), and also the proband's maternal grandmother's specimens. Patient's DNA samples were amplified, fragmented, and hybridized to the Illumina beadchip, followed by single base extension in order to determine the genotype and copy number status for each locus. Results were analysed with BlueFuse Multi version 4.3, using genome reference sequence GRCh37/hg19. The effective resolution was 200 Kb. The hybridization pattern for the patient sample was compared to the patterns from in silico reference sample from the BeadArray cluster file. Deletion or duplication of a genomic segment was detected through a relative change in the hybridization signal from the corresponding probes compared to the reference sample. The sample:expected (reference) signal ratio for each hybridization probe is log_2_ transformed, resulting in a LRR (LogR Ratio). The theoretical LRR for copy number neutral probes is 0.0 (log_2_1 = 0). Heterozygous deletions and duplications have a LRR below 0 and above 0 respectively. Genotyping was performed by examining the B‐allele frequency (BAF), which calculates the percentage B alleles. The theoretical BAF for heterozygous “AB” SNPs is ~0.5 and homozygous “AA” or “BB” SNPs are ~0 or ~1 respectively.

### 2.9 X‐inactivation analysis: SNP microarray method

**Figure 2:**
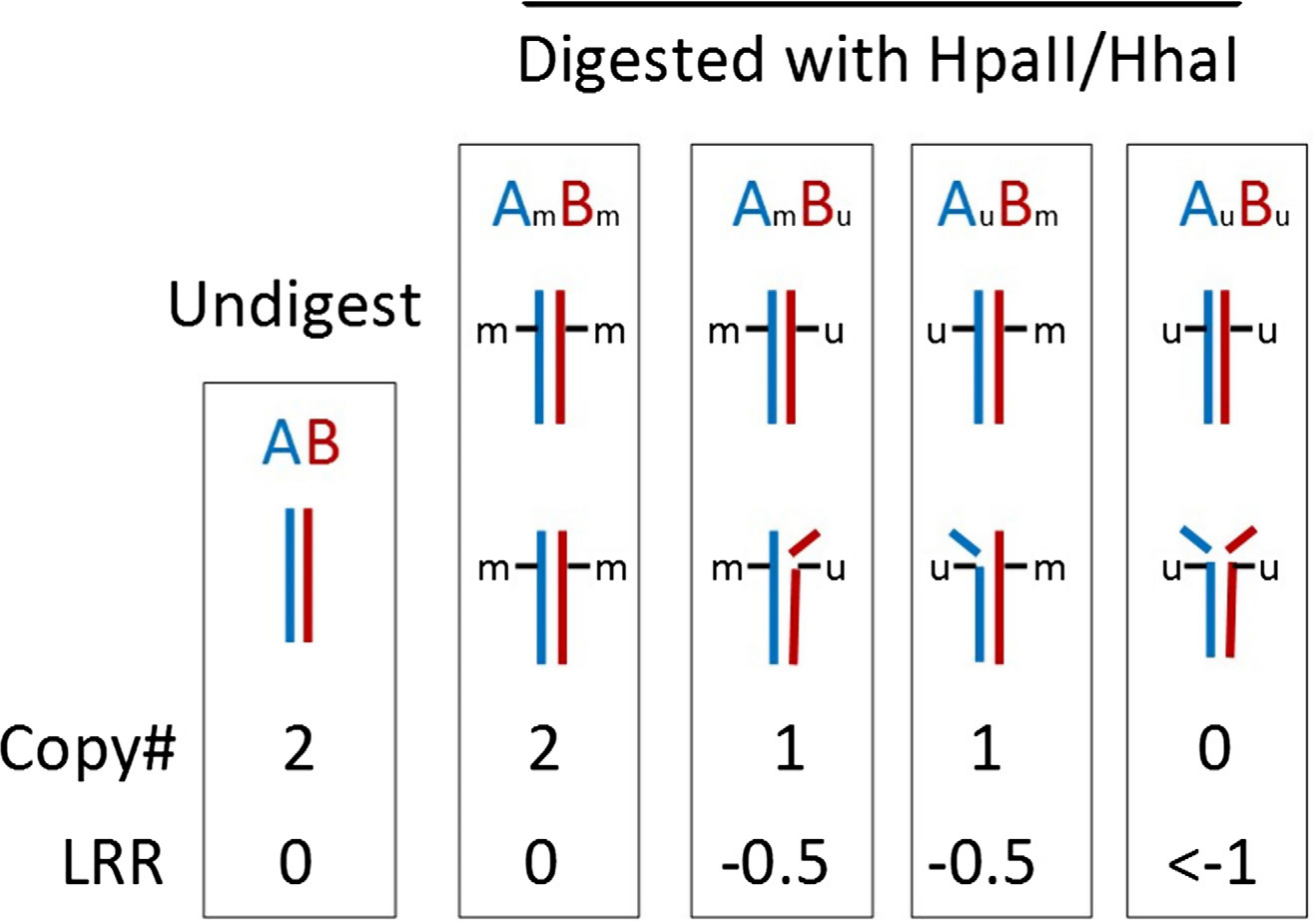
Informative SNPs are heterozygous at the loci (AB), are differentially methylated and demonstrate a LRR of approximately −0.5 post digestion

**Figure 3:**
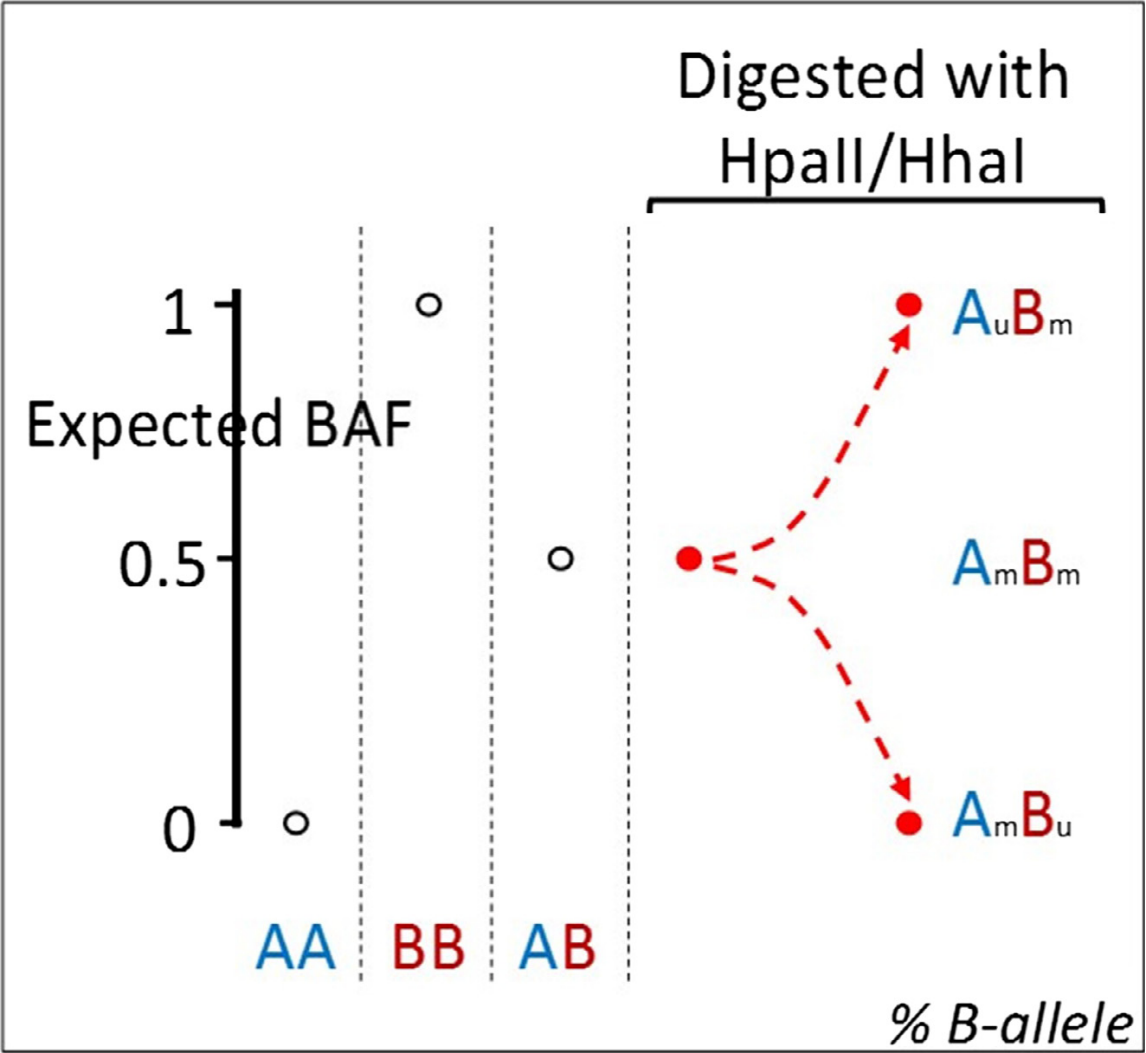
Preferentially methylated (skewed) SNPs have a BAF shift toward 0 or 1

A “proof of concept” method was first devised using informative SNPs for differential methylation status. SNPs were examined in the 15q11.2 Prader‐Willi/Angelman Syndrome critical region (PWACR), within which the maternal allele is known to be preferentially methylated. SNPs within this region were deemed to be informative if they were present in an “AB” allele combination, were differentially methylated and had an LRR of between −0.3 and −1 demonstrating copy number loss after digestion with a methylation‐sensitive restriction enzyme (Figure 2). For each differentially methylated “AB” SNP, the BAF shifted postdigestion toward either 0 or 1, depending on the methylation status of the A and B alleles. Where the A allele was methylated the BAF score was closer to 0. Where the B allele was methylated the BAF score was closer to 1. Where both were methylated the BAF score remained close to 0.5. Where both were unmethylated then the probes did not hybridize and no result was obtained (Figure 3). There were 552 heterozygous SNPs in the proband between 15q11.2 and 15q12. Methylation‐sensitive restriction enzyme digestion was performed (HhaI and HpaII) and the LRR examined, demonstrating that there were 68 SNPs with a LRR between −0.3 and −1 after digest (41 HhaI and 27 HpaII). Following this, SNPs with a significant BAF shift between undigested and digested (between 0 and 0.3 or between 0.7 and 1) were chosen. Eight SNPs showed a significant shift in BAF when comparing undigested versus digested (4 HhaI and 4 HpaII). Three were common SNPs, which demonstrated a BAF shift in both HpaII and HhaI digests. Therefore, in total, there were five heterozygous SNPs whose hybridization to the microarray was affected by allele‐specific differential methylation in the proband. All were mapped within the 133 Kb region at 15q11.2 (nt: 25,068,609‐25,201,659), which is located within the 5‐prime region of the small nuclear ribonucleoprotein polypeptide N (SNRPN) gene and is known to be preferentially methylated on the maternal homolog. Maternal allele‐specific methylation was detected for four of five informative SNPs within this region (Table S1), entirely consistent with the known differential methylation pattern. For the final SNP the parental allele contribution was unable to be determined due to both parents being heterozygous for this SNP.

The identical approach was then taken to select SNPs to study differentially methylated loci on X‐chromosomes. Loci that were AB heterozygous in the proband were selected based on evidence of copy number loss postdigestion (LRR between −0.3 and −1) and a shift in BAF from 0.5 toward either 1 (between 0.7 and 1) or 0 (between 0 and 0.3). This process yielded 21 data points, representing 18 SNPs and identified heterozygous SNPs in the proband that are sensitive to methylation sensitive restriction enzyme digest in an allele‐specific manner.

Informative SNPs were included in further analysis only where there was postdigestion hemizygous loss in the proband's father (loci should be ‘unmethylated’). Three of 21 data points (rs5928367, rs4830643, and rs5945372) were removed from the analysis due to minimal or no change in LRR when comparing the undigested versus digested in the proband's father (Figure S1). The absence of significant change in LRR among males postdigestion could indicate influence by factors independent of X‐chromosome inactivation mediated by methylation, such as incomplete digestion by restriction enzyme and/or differences in DNA sequence (e.g. at the enzyme recognition sites). This left 18 data points (15 SNPs) for further investigation. These SNPs were distributed evenly across the entire X chromosome, with six SNPs in the p‐arm and nine SNPs in the q‐arm.

## 3 RESULTS

### 3.1 Genotype–phenotype correlation

Our proband (Figure 1, V‐7) is the daughter of a male with known severe hemophilia A (Figure 1, IV‐6). She presented at 3 months of age with a history of unusual bleeding including protracted oozing from heel prick blood tests and bruising. Clinical examination did not reveal any specific dysmorphic features and she had normal female genitalia. The family history was relevant for severe hemophilia A in male relatives on the paternal side (Figure 1). The familial *F8* pathogenic variant was known to be a previously described missense mutation F8:c.6545G>A p.(Arg2128His). Female carriers on the paternal side were known to have FVIII levels in the low‐normal to normal range, consistent with expected in the setting of a heterozygous *F8* pathogenic variant.

The proband's mother (IV‐7) and older sister (V‐8) were not known to suffer from any unusual bleeding and there was no history of hemophilia on the maternal side. The proband's mother and maternal grandmother both had a history of a single early first trimester miscarriage (embryonic tissue was not sent for analysis). The maternal grandfather was deceased. The proband had a FVIII level performed on three occasions at diagnosis, with results consistently <0.01 IU/ml. The proband's VWF:Ag (0.86 U/ml) and VWF:RCo (0.87 U/ml) were within normal range and a FVIII inhibitor screen was negative. These results were consistent with a diagnosis of severe hemophilia A in the proband. The proband's mother and sister both had a normal FVIII level (1.11 and 0.61 IU/ml respectively). Her affected father had a FVIII level of <0.01 IU/ml consistent with his known diagnosis of severe hemophilia A. High resolution karyotyping demonstrated that the proband and her mother had a normal 46XX karyotype. *F8* sequencing revealed that the proband carried a single missense pathogenic variant F8:c.6545G>A p. (Arg2128His) which was identical to the pathogenic variant in her father. No *F8* pathogenic variant was identified for the proband's mother. *F8* intron 22 inversion and intron 1 inversion were not detected in the proband or her mother. *F8* MLPA did not demonstrate the presence of a large *F8* gene deletion in the proband or mother. These results demonstrated the absence of a second *F8* pathogenic variant in the proband.

### 3.2 X‐inactivation studies (classical HUMARA method)

**Figure 4:**
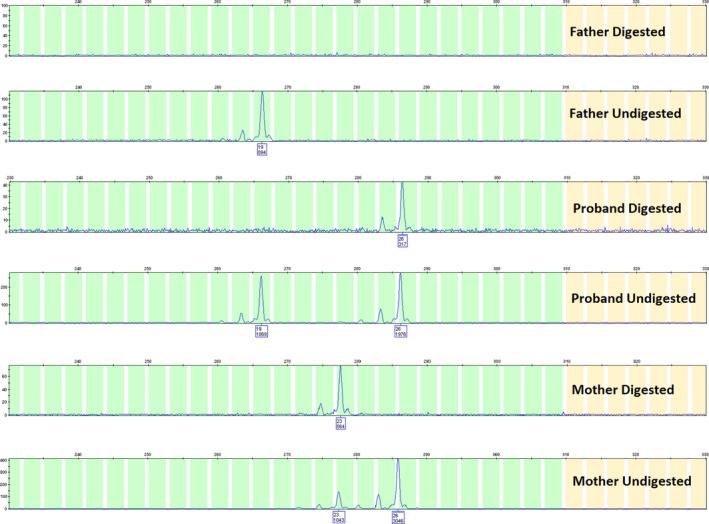
HUMARA X‐inactivation PCR assay results. Row 1: Digested DNA Father, Row 2: Undigested DNA Father, Row 3: Digested DNA Proband, Row 4: Undigested DNA Proband, Row 5: Digested DNA Mother, Row 6: Undigested DNA Mother. Peaks are labeled with CAG repeat size and area under the curve

Prior to methylation‐sensitive restriction enzyme digestion, PCR amplification of the highly polymorphic CAG trinucleotide repeat (exon 1, *AR* gene) demonstrated that the proband had a 19 CAG repeat allele (paternally inherited) and a 26 CAG repeat allele (maternally inherited). The mother demonstrated a 26 CAG allele and a 23 CAG repeat allele. Following methylation sensitive restriction enzyme digest, both the proband and her mother both demonstrated complete disappearance (lack of amplification) of one of their CAG repeats, consistent with 100% skewed X‐chromosome inactivation (Figure 4), however, the proband appeared to completely methylate a different allele (26 CAG repeats) compared to the mother (23 CAG repeats). The proband's maternal grandmother demonstrated a random pattern of methylation (data not shown). A control female specimen that was included in the assay also demonstrated a random pattern of methylation (data not shown).

### 3.3 XIST sequencing

*XIST* sequencing performed in the mother did not demonstrate a pathogenic variant.

### 3.4 X‐inactivation analysis using SNP arrays

The proband's 18 informative data points (representing 15 AB SNPs) were divided into two groups based on the parental allele contribution. There were seven data points where the B allele originated from the father and the A allele originated from the mother (Bpat Amat). There were 11 data points where the A allele originated from the father and B allele originated from the mother (Apat Bmat). Table 1 demonstrates that all seven Bpat Amat data points showed a shift toward the A allele, demonstrating that the maternal allele is methylated and there is skewed X‐inactivation with preferential inactivation of the maternal homolog. Table 2 demonstrates that 11 of 11 Apat Bmat data points showed shift toward the maternal B allele.

**Table 1:** X‐chromosome SNP array in the proband demonstrates a significant BAF shift post digestion toward the maternal (A) allele in all probes, consistent with methylation of the maternal alleles

Probe ID	Proband Genotype	Paternal Genotype	Maternal Genotype	Parental contribution	Digest/Undigest	BAF
rs221931	AB	B	AB	Bpat Amat	Undigest	0.481
					HpaII	0.019
rs5962434	AB	B	AA	Bpat Amat	Undigest	0.546
					HhaI	0.092
rs2298179^a^	AB	B	AB	Bpat Amat	Undigest	0.486
					HhaI	0.016
rs2298179^a^	AB	B	AB	Bpat Amat	Undigest	0.486
					HpaII	0
kgp30607753	AB	B	AB	Bpat Amat	Undigest	0.502
					HpaII	0.037
rs2239470	AB	B	AA	Bpat Amat	Undigest	0.461
					HpaII	0.142
rs915941	AB	B	AA	Bpat Amat	Undigest	0.482
					HhaI	0.011

a SNPs sensitive to both Hhal and Hpall are included as separate data points.

**Table 2:** X‐chromosome SNP array in the proband demonstrates a significant BAF shift post‐digestion toward the maternal (B) allele in all probes, consistent with methylation of the maternal alleles

PROBE ID	Proband Genotype	Paternal Genotype	Maternal Genotype	Parental contribution	Digest/Undigest	BAF
rs12556785	AB	A	AB	Apat Bmat	Undigest	0.53
					HhaI	0.998
rs7063687	AB	A	BB	Apat Bmat	Undigest	0.48
					HpaII	0.975
kgp22761238	AB	A	BB	Apat Bmat	Undigest	0.496
					HpaII	0.967
kgp22758177^a^	AB	A	AB	Apat Bmat	Undigest	0.508
					HhaI	0.996
kgp22758177^a^	AB	A	AB	Apat Bmat	Undigest	0.508
					HpaII	0.969
kgp22824750	AB	A	BB	Apat Bmat	Undigest	0.509
					Hhal	0.817
kgp22741854	AB	A	BB	Apat Bmat	Undigest	0.499
					HpaII	1
rs5937811	AB	A	AB	Apat Bmat	Undigest	0.499
					HhaI	0.989
rs7883362^a^	AB	A	BB	Apat Bmat	Undigest	0.545
					HhaI	0.898
rs7883362^a^	AB	A	BB	Apat Bmat	Undigest	0.545
					HpaII	0.978
kgp30875074	AB	A	BB	Apat Bmat	Undigest	0.537
					HpaII	1

a SNPs sensitive to both Hhal and Hpall are included as separate data points.

In the proband's mother 6 of 15 SNPs (representing 8 data points) were informative (heterozygous). All eight showed significant BAF shift indicative of skewed X‐activation (Table 3). The parental allele contribution for two of these SNPs (rs22758177 and rs2298179) was able to be confirmed, as they were heterozygous (AB) in the mother and homozygous (AA or BB) in the grandmother (Tables 3 and 4). The SNP rs22758177 provided two data points due to its sensitivity to both HpaII and HhaI digests. Both SNPs showed BAF shift consistent with methylation of the allele that was not transmitted from the grandmother. It can therefore be postulated that in the proband's mother, the grandfather's allele is methylated and there is a skewed X‐inactivation with preferential inactivation of the grandfather's homolog.

**Table 3:** X‐chromosome SNP array in the proband's mother demonstrates a significant BAF shift post digestion toward the maternal (B) allele in all probes, consistent with methylation of the maternal alleles

PROBE ID	Maternal Genotype	Digest/Undigest	BAF
rs5937811	AB	Undigest	0.466
		HhaI	0.036
rs221931	AB	Undigest	0.473
		HpaII	0.045
rs2298179^a^	AB	Undigest	0.474
		HhaI	0.045
rs2298179^a^	AB	Undigest	0.474
		HpaII	0.045
rs12556785	AB	Undigest	0.567
		HhaI	0.99
kgp22758177^a^	AB	Undigest	0.54
		HhaI	0.96
kgp22758177^a^	AB	Undigest	0.54
		HpaII	0.96
kgp30607753	AB	Undigest	0.523
		HpaII	0.929

a SNPs sensitive to both Hhal and Hpall are included as separate data points.

**Table 4:** X‐Chromosome SNP array in the proband's maternal grandmother demonstrates no post digestion shift in BAF

PROBE ID	Maternal Grandmother genotype	Digest/Undigest	BAF
rs12556785	AB	Undigest	0.523
		HhaI	0.558
kgp22741854	AB	Undigest	0.504
		HpaII	0.603
rs221931	AB	Undigest	0.516
		HpaII	0.539
rs5962434	AB	Undigest	0.532
		HhaI	0.666
kgp30607753	AB	Undigest	0.507
		HpaII	0.567

In the proband's grandmother 5 of 15 SNPs were informative. All six demonstrated a postdigestion BAF at or very close to 0.5 (Table 4), consistent with random X inactivation.

### 3.5 Meiotic crossover analysis

**Figure 5:**
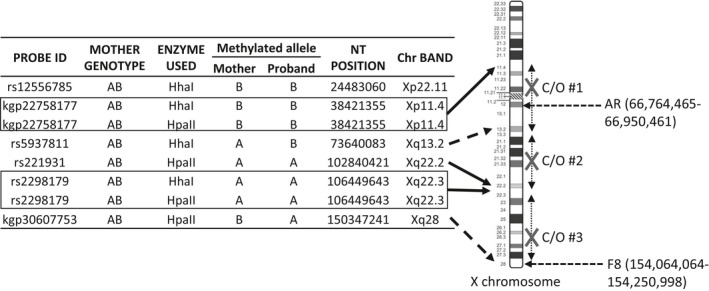
Probable sites for meiotic crossover (M1 C/O) are located between SNPs displaying same allele methylation (solid line) and different allele methylation (dotted line)

The methylated alleles in the mother were compared with the proband to estimate the location of meiotic crossover (C/O) sites. Six SNPs (8 data points) that were heterozygous in both mother and proband were selected to determine if maternal ‘methylated’ or ‘unmethylated’ alleles were transmitted to the proband. Probable crossover sites are located between two SNPs which show different allele methylation between the duos (Figure S2). There were two SNPs (rs5937811 at Xq13.2 and rs30607753 at Xq28) where the methylated alleles differed between the mother and the proband, suggestive of the presence of at least three potential sites for meiotic crossover, estimated to be between Xp11.4 and Xq13.2 (C/O #1), between Xq13.2 and Xq22.2 (C/O #2), and between Xq22.3 and Xq28 (C/O #3) (Figure 5). It is highly probable that the AR gene (targeted by classical X‐inactivation PCR analysis) is located between C/O # 1 and 2.

### 3.6 SNP array

**Figure 6:**
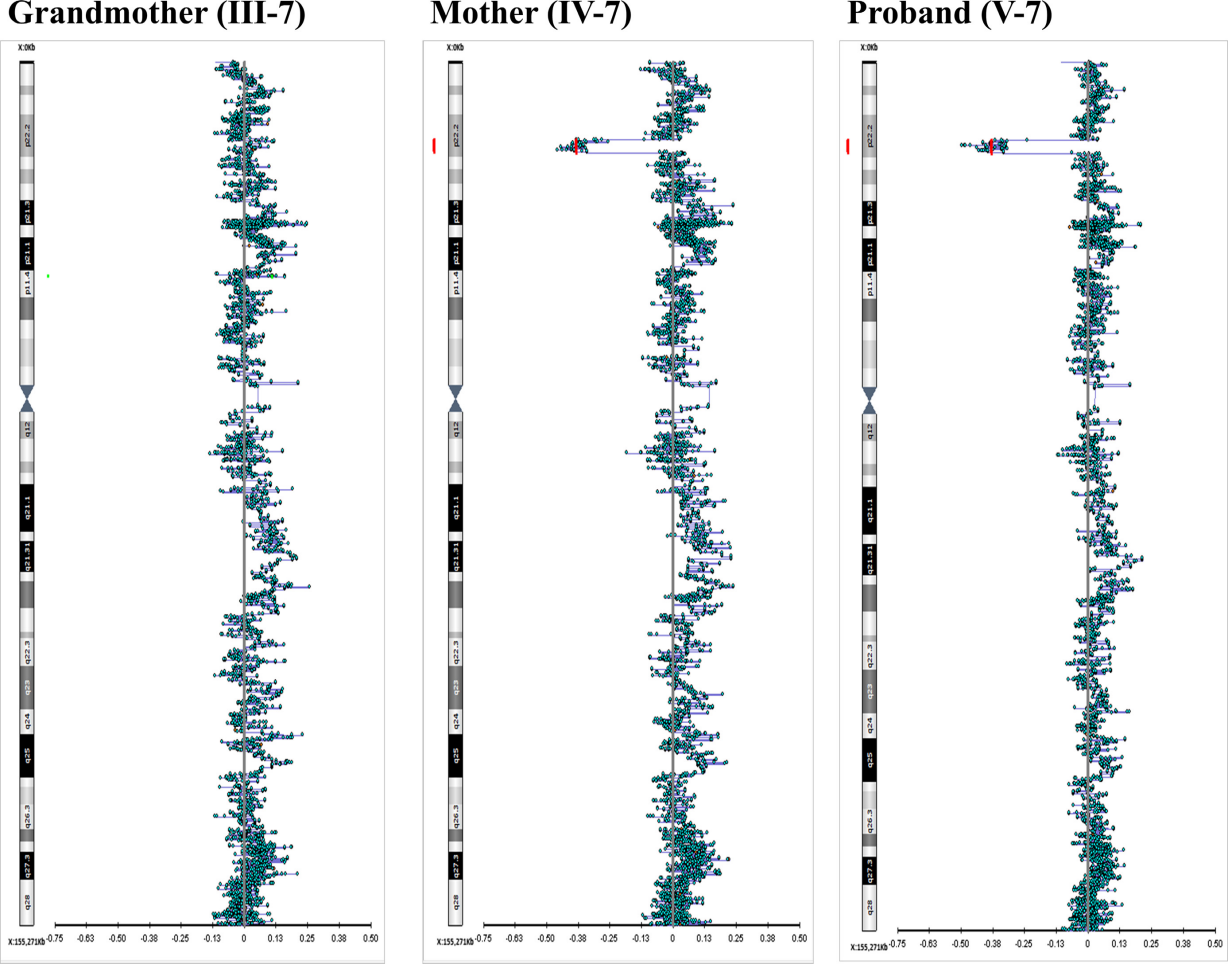
SNP array demonstrating a Xp22.2 microdeletion in the proband (column 3) and her mother (column 2). The microdeletion is not present in the proband's maternal grandmother (column 1)

A heterozygous interstitial deletion of approximately 2.3 Mb was detected at chromosome band Xp22.2 in the proband and her mother, extending from base 13,952,193 to base 16,261,676 (Figure 6). The deletion was not found in the proband's maternal grandmother or father (father not shown). The deleted region contains at least 23 RefSeq genes between *GPM6B* and *MAGEB17*. Of these, three are OMIM listed disease causing genes [*FANCB* (MIM 300515), *PIGA* (MIM 311770), and *AP1S2* (MIM 300629)].

## 4 DISCUSSION

The kindred we have described is segregating a paternally inherited missense *F8* pathogenic variant along with maternally inherited extreme skewing of X‐inactivation. The cytogenetically normal female proband, who is an obligate carrier of the *F8* pathogenic variant, manifests the clinical and laboratory phenotype of severe hemophilia A due to coinheritance of extremely skewed X‐inactivation. There are numerous previous reports of similar cases in the literature (Cau et al., 2006; Dhar, Abramovitz, DiMichele, Gibb, & Gadalla, 2003; Esquilin, Takemoto, & Green, 2012; Orstavik, Orstavik, & Schwartz, 1999; Pavlova et al., 2009), however, the genetic basis for such extreme skewing has rarely been elucidated.

All mammalian female cells undergo X‐inactivation during early embryogenesis, although there is considerable variation between species, and the exact timing of X‐inactivation within the inner cell mass in humans remains uncertain due to the challenges in obtaining suitable tissue for analysis (Belmont, 1996). It has been shown that up to 25% of randomly selected females have mild skewing of X‐inactivation in peripheral blood lymphocytes (ratio >70:30), which conforms to the expected normal distribution model (Allen, Nachtman, Rosenblatt, & Belmont, 1994; Allen et al., 1992; Fialkow, 1973). In general this proportion of skewing appears to correlate with the approximately 20%–25% of females with a heterozygous *F8* pathogenic variant who have a mildly reduced FVIII level (Byams et al., 2011). Nonetheless it must be highlighted that the X‐inactivation pattern in peripheral blood lymphocytes, in which *F8* is not expressed, does not always readily explain the FVIII levels observed in individual carriers of hemophilia A (Orstavik, Scheibel, Ingerslev, & Schwartz, 2000). On a general population level divergent results have been obtained when X‐inactivation patterns were compared between peripheral blood, skin and muscle (Gale, Wheadon, Boulos, & Linch, 1994). It is a reasonable assumption that similar disparity exists between peripheral blood lymphocytes and the liver, and that tissue specific liver based skewing has a greater contribution toward variation in FVIII levels observed between carriers, although this is clearly not feasible to study on a population level.

More pronounced skewing of X‐inactivation (>90:10) can be demonstrated in up to 10% of phenotypically normal females (Gale et al., 1994; Nance, 1964; Naumova et al., 1996; Plenge et al., 1997), however, extreme skewing (>95:5) is rare (<1% of female newborns) (Amos‐Landgraf et al., 2006). The probability of finding skewing to this degree in a mother and daughter due to chance alone is exceptionally low, and we would postulate that most such cases are likely to have a genetic basis. “Extreme” skewing has been variably defined in the literature, but generally refers to the marked nonrandom inactivation of either the paternally or maternally derived X‐chromosome in the majority of cells. In females who do not harbor an X‐linked disease‐causing variant on the active chromosome such extreme skewing is clinically silent (Allen et al., 1994). Females who are also heterozygous for an X‐linked recessive disorder will manifest features of the disorder if the active X‐chromosome has the disease‐causing variant. In our kindred the extremely skewed X‐inactivation allowed a heterozygous *F8* pathogenic variant on the active X‐chromosome to become clinically manifest as severe hemophilia A.

Pegoraro et al. (1997) reported the largest characterized kindred manifesting extreme skewing of X‐inactivation as a familial trait. This five‐generation kindred was originally identified due to an affected female with Duchenne muscular dystrophy (DMD; MIM 310200) in whom the maternally derived X‐chromosome was inactivated in >95% of cells. Further investigation determined that 16 of 50 female pedigree members showed extremely skewed (≥95:5) X‐chromosome inactivation, including the proband's mother. Similar levels of skewing were not observed in any of the unrelated female controls. Linkage analysis localized the trait to Xq28, and subsequent FISH analysis revealed a relatively large deletion (~800 kb) at this locus. There was complete segregation of this deletion with female carriers manifesting highly skewed X‐inactivation, with the paternally derived X‐chromosome active in >95% of cells. Females carrying the deletion also had a statistically significant (*p* < .02) increased rate of spontaneous abortion (40%) compared with their siblings who did not carry the deletion (15%). The authors hypothesized that the Xq28 deletion caused a survival disadvantage to cells in which the affected X‐chromosome was active, leading to loss of hemizygous male embryos that inherited the deletion, and extreme skewing of X‐inactivation in heterozygous females (Pegoraro et al., 1997).

Parolini et al. (1998) described a similar but much smaller kindred in which Wiskott‐Aldrich syndrome, an X‐linked recessive hematological disorder, was diagnosed in an 8‐year‐old girl caused by a sporadic pathogenic variant in the *WAS* gene on the paternally derived X‐chromosome, with associated extreme skewing of X‐inactivation (maternal X‐chromosome inactive in >95% cells). This case was particularly remarkable in that female carriers of *WAS* pathogenic variants typically have tissue‐specific extreme skewing of X‐inactivation in hematopoietic cells that favors the nonmutated X‐chromosome, presumably due to a survival disadvantage mechanism (the WAS protein is only expressed in hematopoietic cells, and is postulated to have an important role in cytoskeletal function). The proband's mother and maternal grandmother also had extremely skewed X‐inactivation, unfortunately the genetic basis for familial skewing was not identified (Parolini et al., 1998).

Extremely skewed X‐inactivation as a result of gene variants causing a survival disadvantage has been described in association with a number of other severe X‐linked recessive hematological conditions (Favier et al., 2000). Negative selection in early embryogenesis against cells bearing a mutant allele has been described in heterozygote female carriers of X‐linked Fanconi anemia (*FANCB,* MIM 300514) (Meetei et al., 2004), Bruton agammaglobulinemia (*BTK,* MIM 300755) (Allen et al., 1994), severe combined immunodeficiency syndrome (*IL2RG,* MIM 300400) (Puck, Nussbaum, & Conley, 1987), and dyskeratosis congenita (*DKCX,* MIM 305000) (Devriendt et al., 1997). Historically these families have generally been studied on the basis of having males with a clinically recognized syndrome, and the presence of extremely skewed X‐inactivation is utilized to readily identify female carriers, even in the absence of an identifiable disease‐causing variant. It must be reiterated that such female carriers, although extremely skewed, are generally asymptomatic unless they coinherit another X‐linked disorder.

Female heterozygote carriers of these smaller X‐chromosome deletions or nonsense variants that cause marked skewing may have 50% of their male children demonstrate a recognizable syndrome (e.g. Fanconi anaemia) or various nonspecific congenital abnormalities. For example (Pavlova et al., 2009), described a female patient with severe hemophilia A with extremely skewed X‐inactivation who had two brothers with Coffin Lowry syndrome (a severe X‐linked recessive neurodevelopmental disorder; resulting from a nonsense variant in exon 5 of the *RSK2* gene at Xp22.2). The affected female had skewed X‐inactivation due to being a carrier of the *RSK2* pathogenic variant (maternally derived X‐chromosome inactivated in >95% cells) as well as a de novo paternally derived germline pathogenic variant in exon 25 of the *F8* gene (Pavlova et al., 2009).

The 2.3 Mb deletion identified at Xp22.2 in our kindred has not been previously reported. At least three disease‐associated genes (*FANCB*,* AP1S2,* and *PIGA*) were identified within the deleted region. A search of DECIPHER found two previous reports of male patients with X‐chromosome deletions that overlapped in part with our proband's deleted region at Xp22.2. A male infant (DECIPHER ID 256757) was reported with a 531 kb deletion that involved *FANCB* and had cardiac defects (ventricular septal defect), trachea‐esophageal fistula, renal hypoplasia, and skeletal abnormalities. A second male infant (DECIPHER ID 2376) was reported with a 2.88 Mb deletion that involved *AP1S2* and was severely affected with intellectual impairment, a seizure disorder, cataracts, Tetralogy of Fallot, and muscular hypotonia (Firth et al., 2009). The reported germline *PIGA* mutation spectrum for surviving severely affected human males comprises four missense variants, one nonsense variant, one frameshift variant, and one small in frame deletion (Tarailo‐Graovac et al., 2015). No large deletions involving *PIGA* in surviving humans are reported, and studies in mice have revealed that complete disruption of the *PIGA* gene results in early embryonic lethality in males (Nozaki et al., 1999).

We postulate that the 2.3 Mb Xp22.2 deletion identified in our kindred arose de novo in the proband's mother (on the grandfather's homolog). Given the size of the deletion (affecting the critical *PIGA* gene) and that large *PIGA* deletions produce early embryonic lethality in male mice we hypothesize that it would produce extreme skewing of X‐inactivation via a “cell lethal” mechanism rather than a survival disadvantage mechanism, resulting in early embryonic nonviability of male zygotes. If true, this obviously has considerable implications for genetic counseling in this family: 50% of male zygotes conceived by the proband's parents would be nonviable, and 50% of females would be affected by severe hemophilia A; all the proband's future male offspring would have severe hemophilia A, as male zygotes with the microdeletion would be nonviable. Given the frequency with which early embryonic loss is encountered, along with the presumably unimpaired ability to conceive female infants, it is unlikely that females with X‐chromosome microdeletions of this nature would come to medical attention unless they have a female child who coinherits (and therefore manifests) another X‐linked disorder such as hemophilia.

Classical X‐inactivation analysis targets a highly polymorphic triplet repeat region at a single locus (within exon 1 of the *AR* gene) (Allen et al., 1992) Interpretation can be complicated by meiotic crossover events which occur commonly on the X‐chromosome (Tease, Hartshorne, & Hulten, 2002). To overcome this problem in our kindred we developed a novel method for X‐inactivation analysis by examining differential methylation patterns in multiple informative X‐chromosome SNPs. This method involved the use of probes distributed along the entire length of the X‐chromosome, thereby allowing recombination sites to be reasonably predicted. The demonstration that the *AR* receptor gene is located between two of the probable meiotic crossover sites explains the unexpected finding on classical X‐inactivation studies. It should be highlighted that our method has not yet been trialled in additional control families (both skewed and nonskewed), nor has it been validated to provide quantitation regarding degree of skewing. However, the method demonstrates a novel multitarget approach to X‐inactivation analysis that overcomes the issue of recombination and clearly warrants further exploration of its potential utility in routine diagnostic practice.

We propose that trio‐SNP analysis, now routinely available in commercial diagnostic laboratories, should be utilized in all cases in which extreme skewing of X‐inactivation is suspected or confirmed. Although a previous case–control study (45 cases and 45 controls) argued against the view that microarray‐detectable abnormalities of the X‐chromosome are a frequent cause of nonrandom skewing, it must be highlighted that their definition of “highly skewed” X‐inactivation was only ≥85%, and therefore it is likely that there was significant overlap of cases with random stochastic skewing erroneously included in the highly skewed group. Interestingly the authors did find one female with extremely skewed X‐inactivation (>95%) in blood and left and right buccal smears who had a 5.5‐Mb del(X)(p22.2p22.1) (Jobanputra et al., 2012)

In conclusion, we utilized a combination of classical X‐inactivation analysis (targeting the *AR* gene) and case‐parent trio SNP microarray analysis to confirm familial extreme skewing of X‐inactivation, and to identify a maternally derived Xp22.2 microdeletion as the likely mechanism. Our investigation was complicated by the initial HUMARA assay finding that the mother and proband had >95% methylation of divergent CAG alleles and a recombination event was suspected. We subsequently demonstrated a high likelihood of recombination involving the *AR* gene using a novel modification of existing SNP array methods utilizing differentially methylated SNPs, with analysis before and after digestion with a methylation‐sensitive restriction enzyme. We believe this represents the first reported use of SNP technology in this context. Our opinion is that true “extreme” nonrandom skewing (≥95:5) is a rare occurrence, but when defined correctly there is a high probability of finding an X‐chromosome disease‐causing variant or larger deletion resulting in X‐inactivation through a survival disadvantage or cell lethal mechanism. We propose that for females with unexplained severe phenotypic expression of an X‐linked recessive disorder trio‐SNP microarray should be undertaken in combination with X‐inactivation analysis. In the absence of a demonstrable microdeletion whole exome sequencing of the X chromosome should be considered to exclude pathogenic variants.

## CONFLICT OF INTEREST

None declared.
