# Supplementary figures and images for: Demonstration of a novel Xp22.2 microdeletion as the cause of familial extreme skewing of X‐inactivation utilizing case‐parent trio SNP microarray analysis
Source: Mol Genet Genomic Med. 2018 Feb 28;6(3):357–69. doi: 10.1002/mgg3.378 (PMC6014479; doi:10.1002/mgg3.378)

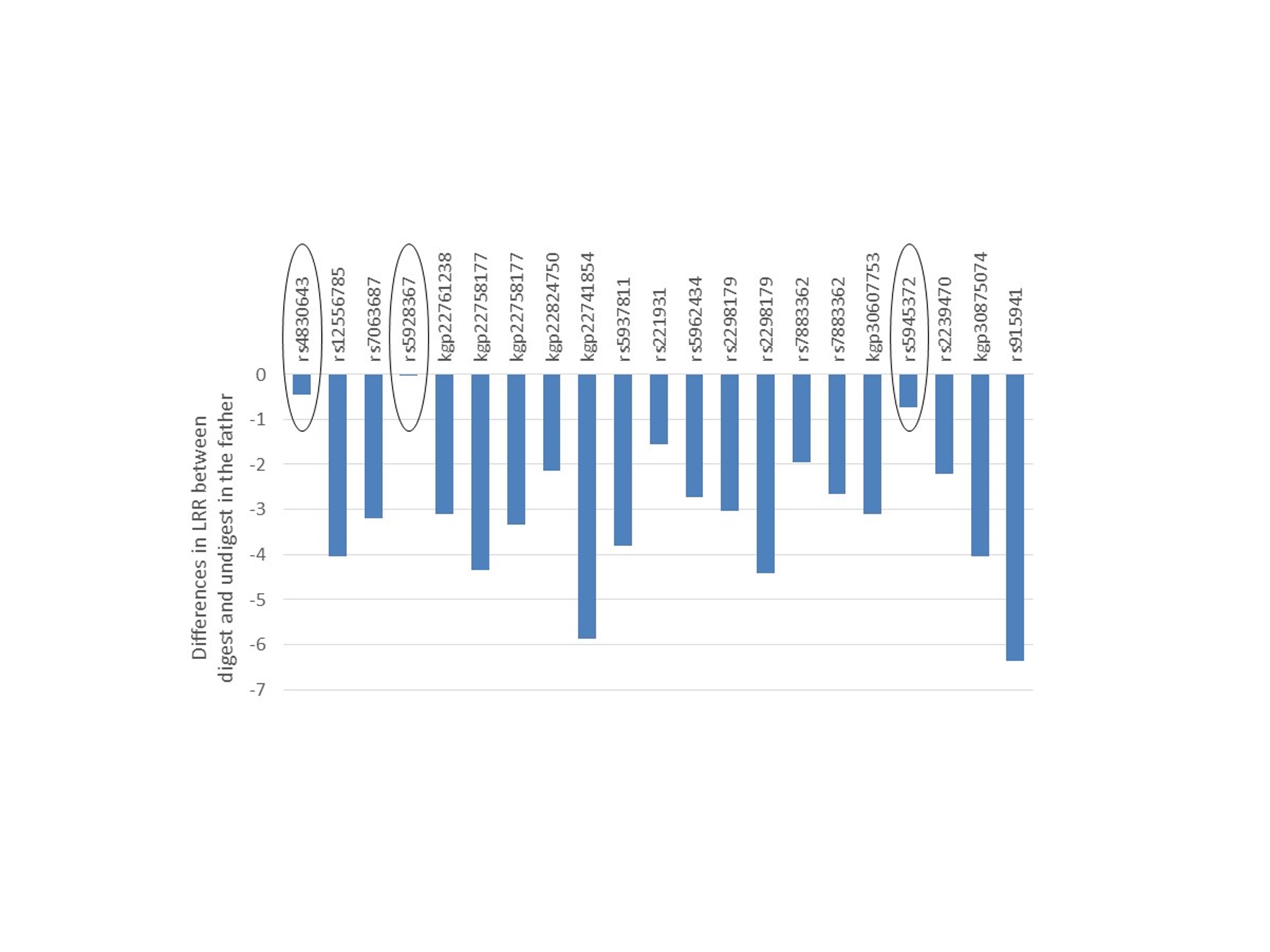

Supplement: Supplementary file 1 [file MGG3-6-357-s001.jpg]

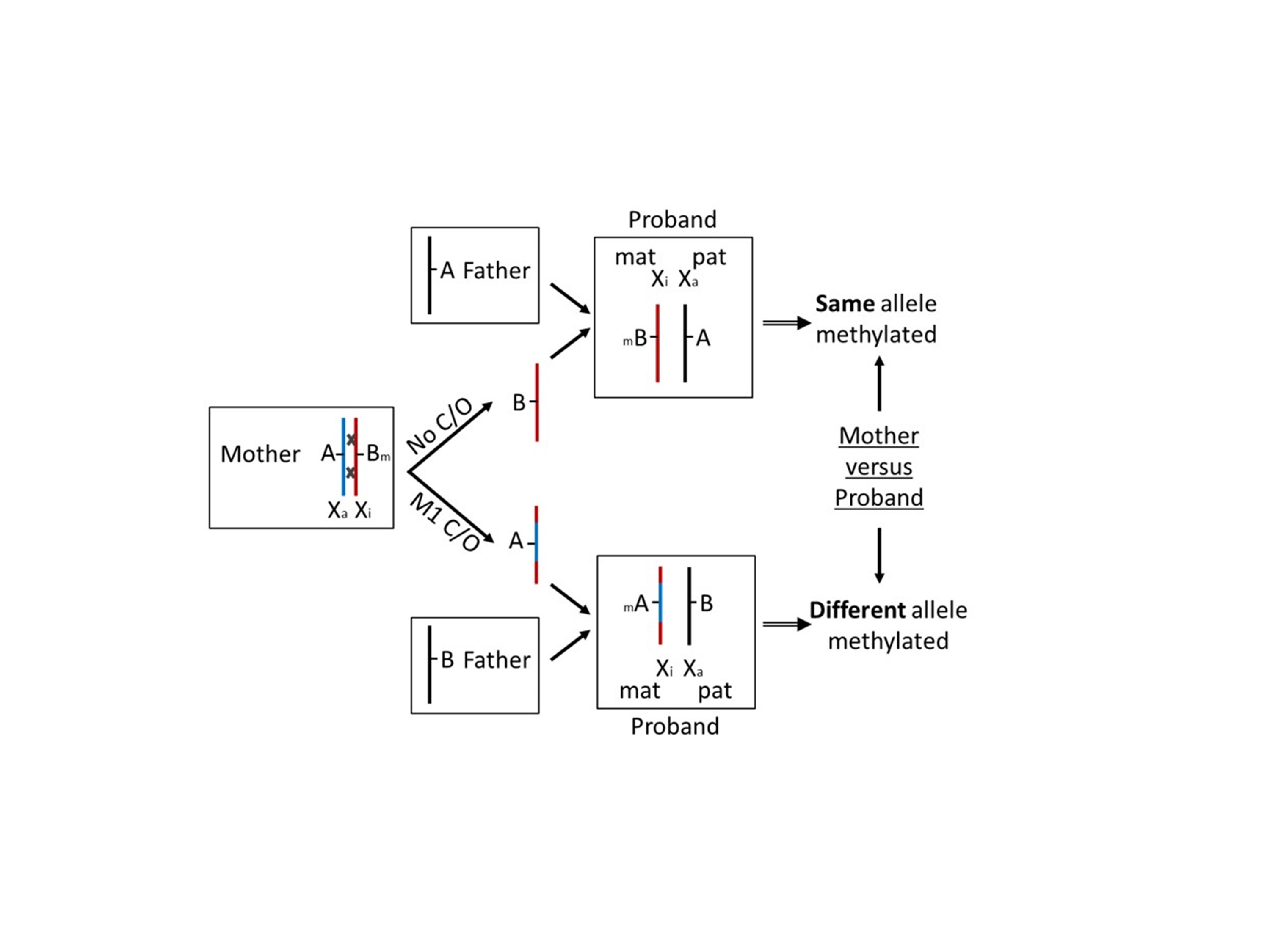

Supplement: Supplementary file 2 [file MGG3-6-357-s002.jpg]
