# Supplementary material for: Demonstration of a novel Xp22.2 microdeletion as the cause of familial extreme skewing of X‐inactivation utilizing case‐parent trio SNP microarray analysis
Source: Mol Genet Genomic Med. 2018 Feb 28;6(3):357–69. doi: 10.1002/mgg3.378 (PMC6014479; doi:10.1002/mgg3.378)
Supplement: Supplementary file 3 [file MGG3-6-357-s003.docx]

|  |  |  |  |  |  |  |  |
| --- | --- | --- | --- | --- | --- | --- | --- |
| Probe ID | Proband | Pat^a^ | Mat^b^ | Parental contribution | Digest | BAF | Comment |
| rs17772751 | AB | AB | AA | Bpat Amat | Undigest | 0.474 | Shift towards A *A (maternal) allele methylated* |
|  |  |  |  |  | HhaI | 0.153 |  |
| rs11633270 | AB | AB | AA | Bpat Amat | Undigest | 0.466 | Shift towards A *A (maternal) allele methylated* |
|  |  |  |  |  | HpaII | 0.154 |  |
|  |  |  |  |  | HhaI | 0.214 |  |
| rs2167926 | AB | AB | AA | Bpat Amat | Undigest | 0.468 | Shift towards A *A (maternal) allele methylated* |
|  |  |  |  |  | HpaII | 0.251 |  |
|  |  |  |  |  | HhaI | 0.239 |  |
| rs2647354 | AB | AB | AA | Bpat Amat | Undigest | 0.511 | Shift towards A *A (maternal) allele methylated* |
|  |  |  |  |  | HpaII | 0.362 |  |
| rs4906940 | AB | AB | AB | Undetermined | Undigest | 0.532 | Shift towards A A allele methylated *Parental allele contribution not determined* |
|  |  |  |  |  | HpaII | 0.032 |  |
|  |  |  |  |  | HhaI | 0 |  |
|  |  |  |  |  |  |  |  |

Supp. Table S1. SNP probes which target 15q11.2 demonstrate a BAF shift post digestion consistent with expected differential methylation. ^a^Pat = Paternal. ^b^Mat = Maternal.
